# Neglected Proximal Tibiofibular Joint Dislocation Treated With a Cortical Button Suspension Device: A Case Report

**DOI:** 10.7759/cureus.45284

**Published:** 2023-09-15

**Authors:** Takeo Mammoto

**Affiliations:** 1 Department of Orthopedic Surgery and Sports Medicine, Tsukuba University Hospital Mito Kyodo General Hospital, Mito, JPN

**Keywords:** cortical button suspensory device, surgical procedure, proximal tibiofibular joint, dislocation, knee joint

## Abstract

Proximal tibiofibular joint dislocation is a rare knee injury. Hence, its diagnosis is often missed. Herein, we have reported a case of posterior lateral proximal tibiofibular joint dislocation that was initially missed because it was associated with a fibula diaphyseal fracture. Our patient was a 23-year-old male with a complaint of left lateral knee pain and a history of fall from a motorcycle. He was treated with a cortical button suspension device. The patient reported no symptoms or complications at the one-year follow-up. Proximal tibiofibular joint dislocation is easily neglected if not considered as a diagnosis during clinical assessment. Half of these cases present with symptoms such as chronic pain and peroneal nerve palsy that require surgical treatment. A detailed physical examination and close review of imaging findings are important to establish a definitive diagnosis. A cortical bone button suspension device could be the appropriate treatment for cases requiring surgical management.

## Introduction

Proximal tibiofibular joint dislocation is a very rare knee injury, accounting for only 1% of all knee injuries [1-3]. These injuries are most commonly caused by direct, high-energy trauma, often from sports activities or traffic accidents. The diagnosis of proximal tibiofibular joint dislocation can be difficult to establish and is often missed during initial treatment. It can be easily overlooked even on a plain radiograph examination of the knee [4]. However, early detection and prompt treatment are essential to prevent further complications such as chronic pain and peroneal nerve palsy [4].

Although the optimal treatment for proximal tibiofibular joint dislocation has not yet been established, closed reduction could be considered as a first treatment option. If closed reduction is unsuccessful, open reduction should be performed. Recently, cortical button suspension device fixation is increasingly being used to stabilize proximal tibiofibular joint dislocations [5-7]. Herein, we report a case of neglected proximal tibiofibular joint dislocation that was initially missed because it was associated with a diaphyseal fracture of the fibula. It was managed with a cortical button suspension device.

## Case presentation

A 23-year-old man presented to our hospital with the chief complaint of left lateral knee pain. He had been injured during a fall while riding a motorcycle four weeks prior and had gone to a nearby emergency department. Based on physical and radiographic findings during the initial visit, he was diagnosed with a diaphyseal fracture of the fibula and was managed with joint immobilization and a splint for two weeks. Two weeks after the injury, he was allowed to walk. However, the patient continued to experience knee pain, leading him to present to our hospital.

**Figure 1:**
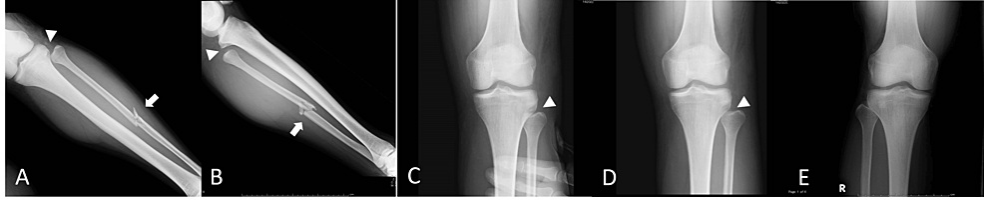
Initial X-ray radiographs and fluoroscopic findings (A, B) Initial X-ray radiographs (anteroposterior (A) and lateral view (B)). A fracture at the fibular diaphysis is diagnosed (white arrow), but the dislocation of the proximal tibiofibular joint is neglected (white arrowhead). (C, D, E) Fluoroscopic findings. Under fluoroscopic guidance, the dislocated fibula head of the left knee is reduced manually (C); however, it is easily re-dislocated (D); right knee anteroposterior view (E).

During this visit, physical examination revealed swelling on the anterolateral side of the knee joint, and tenderness and abnormal mobility of the fibular head. The shuck test, a palpation test for anterior translation of the fibular head relative to the tibia, was positive when an anterior translation of the fibular head relative to the tibia was palpable, often with a clunk [5,8]. In this case, the anterior translation of the fibular head relative to the tibia was grossly visible and palpable. No abnormal neurological findings were noted, and there was no evidence of knee joint instability. Radiographs showed a fracture of the fibular diaphysis and a posterior lateral dislocation of the proximal tibiofibular joint (Figure 1 A, B).

**Figure 2:**
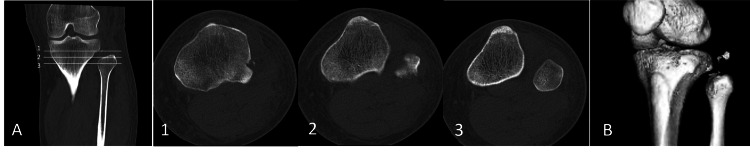
Computed tomography images (A) Computed tomography images of axial (1-3) and (B) 3D reconstruction images. The computed tomography image reveals a posterior lateral proximal tibiofibular dislocation with small bony fragments.

The computed tomography image revealed a posterior lateral proximal tibiofibular dislocation with small bony fragments (Figure 2 A,B).

The patient was finally diagnosed with a posterior lateral proximal tibiofibular joint dislocation that was associated with a fibular diaphyseal fracture. Fluoroscopic examination demonstrated that the dislocated fibular head could be manually reduced. However, it was easily re-dislocated (Figure 1 C, D, and E). Given that more than four weeks had passed since the injury and the patient was still experiencing symptoms such as pain, the decision was made to perform surgical management.

The procedure was performed under general anesthesia. After the induction of anesthesia, a thorough knee examination was done, including an anterior-posterior shuck test for the proximal tibiofibular joint. It was positive as anterior translation of the fibular head relative to the tibia was palpated with a clunk.

**Figure 3:**
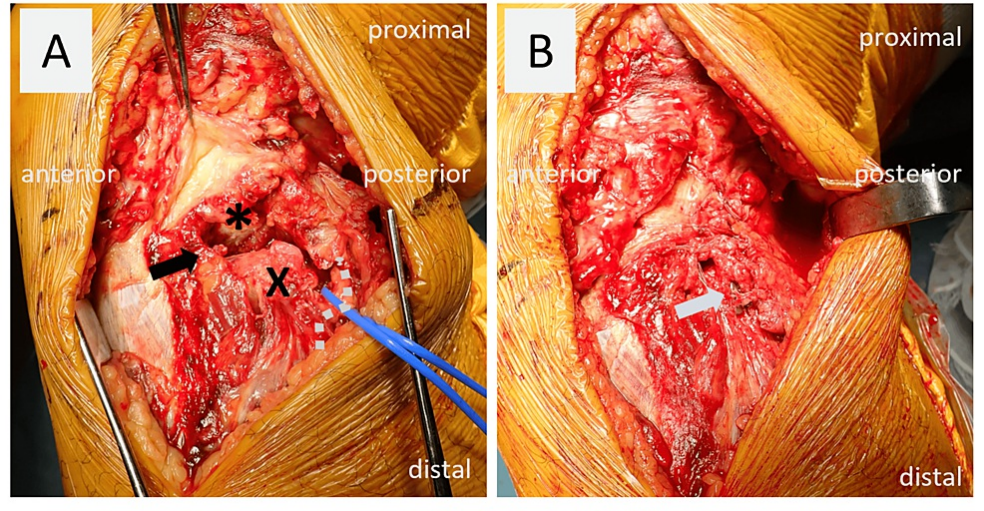
Intraoperative findings (A, B) On gross examination, the joint is observed to be dislocated posterolaterally (A, black arrow). The articular surface of the tibial side in the proximal tibiofibular joint (*), fibular head (x), and common peroneal nerve (white dot) are observed with a vessel loop passed around it. The joint dislocation is reduced after the placement of a cortical button suspension device (B, white arrow).

During the surgery, initially, the diaphysis of the fibula was fixed with a one-third tubular plate (LCP, Synthes). Fixation of the fibular diaphyseal fracture was done because the proximal fragment was displaced distally, and the length of the fibula was shortened. After fixing the fibular diaphysis, the posterolateral dislocation and instability of the proximal tibiofibular joint remained. Then a posterior incision was made over the head of the fibula to fix the proximal tibiofibular joint. In this case, direct visualization of the injury site was deemed necessary to remove hematomas and scarring for successful repair. Careful dissection was performed, and the common peroneal nerve was identified. The nerve was carefully protected inferiorly and dissected, exposing the proximal tibiofibular joint (Figure 3).

**Figure 4:**
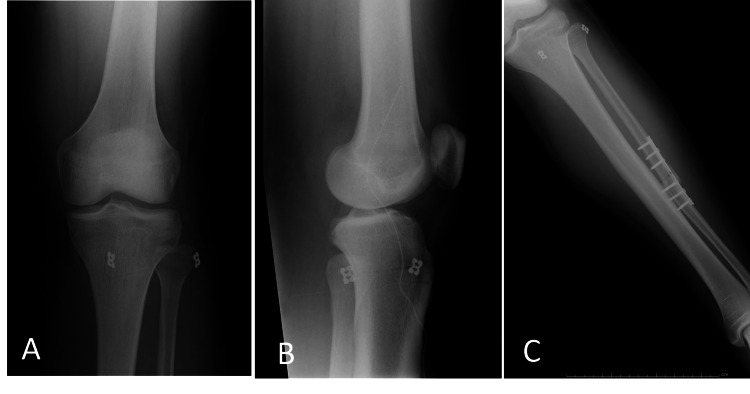
Postoperative X-ray radiographs (A, B, C) Postoperative X-ray radiographs (anteroposterior (A) and lateral view (B) and anteroposterior view of the leg (C)). Dislocation of the proximal tibiofibular joint is reduced.

The metaphyseal thickening of the fibula was identified for the deployment of the cortical button suspensory fixation device (Tight-Rope, Arthrex). Under fluoroscopic guidance, a guide pin was inserted from the posterolateral side of the fibular head, targeting the anteromedial surface of the tibia, just medial to the tibial tuberosity. The angle was approximately 30° from posterolateral to anteromedial to avoid compromising the posterior tibial cortex [5,6]. After confirming the appropriate placement of the guide pin, a 4.0 mm cannulated drill bit was used to drill over it. The drill and guide pin were then removed, and the adjustable loop was advanced using a shuttle suture. A cortical button was applied once the adjustable loop emerged from the medial tibia. By applying gentle pressure under the lateral button, the sutures were alternately pulled to shorten the adjustable loop structure and secure the lateral button to the fibula (Figure 3). The implant was positioned such that the joint dislocation was reduced at 60 degrees of knee flexion. A second shuck test was performed to confirm that the abnormal mobility of the tibiofibular joint had been eliminated following device fixation (Figure 4).

Postoperatively, the patient was managed with knee joint extension immobilization and permitted toe-touch weight-bearing for two weeks. After two weeks, weight-bearing was gradually increased, and by six weeks, full weight-bearing was allowed. Passive range of motion was permitted up to 90 degrees, progressing to full range of motion after six weeks. The patient returned to playing soccer five months post-surgery. At the one-year follow-up, the patient had returned to daily life and sports activities without any reported symptoms, including chronic knee pain and discomfort from the device.

## Discussion

The most significant finding of this study is that a proximal tibiofibular joint dislocation can easily be overlooked if not considered during clinical assessment. A comprehensive physical examination and a careful review of imaging findings are crucial for establishing a definitive diagnosis.

This case report outlines an instance of proximal tibiofibular joint dislocation that was neglected due to its concurrent fibula diaphyseal fracture. It was managed surgically using a cortical button suspension device.

Proximal tibiofibular joint dislocation is rare, accounting for an estimated 1% of all knee injuries [6]. It can be overlooked in clinical practice if joint dislocation is not part of the differential diagnosis during the assessment. Although the optimal treatment for this condition has not yet been established, nonoperative treatment could be the first line of intervention.

After one to six weeks of nonoperative management using long-leg cast immobilization, 59% of patients improved without residual symptoms. The remaining patients experienced ongoing symptoms [8]. Nonoperative treatment combined with physical therapy results in 50% of patients retaining residual instability in the proximal tibiofibular joint, thus requiring surgical stabilization [9].

Various surgical techniques have been documented, including fibular head resection, joint fixation, and reconstruction [5-8,10]. While no instability symptoms are observed following fibular head resection, the risk of peroneal nerve palsy remains [9,11]. Techniques like K-wire or screw fixation have been employed in cases featuring chronic recurrent dislocation and symptomatic subluxation [8,12]. These procedures often necessitate the subsequent removal of the implants because they rigidly fixate the proximal tibiofibular joint and restrict its normal physiological movements like external rotation and anterior-posterior translation of the fibula [4].

Reconstructive methods include free grafts and cortical button suspensory devices [5-8,10]. No symptoms of instability have been reported with free graft reconstruction. However, complications such as perioperative fractures and donor site morbidity from autologous tendon harvesting exist [8].

Cortical suspensory fixation devices are increasingly utilized for ligamentous structure repair, particularly in the distal syndesmosis [13]. These devices have demonstrated mechanical superiority to screw fixation, and favorable clinical results have been observed. They also carry a lower risk of intraoperative fractures during drilling compared with free graft construction [13]. These devices enable improved physiological movement of the proximal tibiofibular joint, reducing the need for hardware removal due to restricted joint movements [5-7]. While there is concern over peroneal nerve symptoms with this device, the risk is deemed comparable to that associated with screw and K-wire fixation, as well as free graft reconstruction.

## Conclusions

We have reported a case of proximal tibiofibular joint dislocation that was overlooked due to an associated fibula diaphyseal fracture. This case was managed surgically using a cortical button suspension device. Proximal tibiofibular joint dislocation can easily be neglected if not considered during the assessment. In approximately half of these cases, symptoms such as chronic pain and peroneal nerve palsy necessitate surgical intervention. When joint dislocation is suspected, a thorough physical examination and meticulous review of imaging findings are crucial. Cortical button suspensory devices may offer a suitable treatment option for cases requiring surgical management.
